# Effectiveness of imaging modalities for screening IgG4-related dacryoadenitis and sialadenitis (Mikulicz’s disease) and for differentiating it from Sjögren’s syndrome (SS), with an emphasis on sonography

**DOI:** 10.1186/s13075-015-0751-x

**Published:** 2015-08-23

**Authors:** Mayumi Shimizu, Kazutoshi Okamura, Yoshitaka Kise, Yohei Takeshita, Hiroko Furuhashi, Warangkana Weerawanich, Masafumi Moriyama, Yukiko Ohyama, Sachiko Furukawa, Seiji Nakamura, Kazunori Yoshiura

**Affiliations:** Department of Oral and Maxillofacial Radiology, Kyushu University Hospital, 3-1-1 Maidashi, Higashi-ku, Fukuoka 812-8582 Japan; Department of Oral and Maxillofacial Radiology, Faculty of Dental Science, Kyushu University, 3-1-1 Maidashi, Higashi-ku, Fukuoka 812-8582 Japan; Department of Oral and Maxillofacial Radiology, School of Dentistry, Aichi Gakuin University, 1-100 Kusumoto-cho, Chikusa-ku, Nagoya 464-8650 Japan; Section of Oral and Maxillofacial Oncology, Division of Maxillofacial Diagnostic and Surgical Sciences, Kyushu University, 3-1-1 Maidashi, Higashi-ku, Fukuoka 812-8582 Japan; Section of Oral and Maxillofacial Surgery, Division of Maxillofacial Diagnostic and Surgical Sciences, Kyushu University, 3-1-1 Maidashi, Higashi-ku, Fukuoka 812-8582 Japan

## Abstract

**Introduction:**

The aim of this study was to clarify the effectiveness of various imaging modalities and characteristic imaging features in the screening of IgG4-related dacryoadenitis and sialadenitis (IgG4-DS), and to show the differences in the imaging features between IgG4-DS and Sjögren’s syndrome (SS).

**Methods:**

Thirty-nine patients with IgG4-DS, 51 with SS and 36 with normal salivary glands were enrolled. Images of the parotid and submandibular glands obtained using sonography, 2-[^18^F]-fluoro-2-deoxy-D-glucose positron emission tomography/computed tomography (FDG-PET/CT), computed tomography (CT) and magnetic resonance imaging (MRI) were retrospectively analyzed. Six oral and maxillofacial radiologists randomly reviewed the arranged image sets under blinded conditions. Each observer scored the confidence rating regarding the presence of the characteristic imaging findings using a 5-grade rating system. After scoring various findings, diagnosis was made as normal, IgG4-DS or SS, considering all findings for each case.

**Results:**

On sonography, multiple hypoechoic areas and hyperechoic lines and/or spots in the parotid glands and obscuration of submandibular gland configuration were detected mainly in patients with SS (median scores 4, 4 and 3, respectively). Reticular and nodal patterns were observed primarily in patients with IgG4-DS (median score 5). FDG-PET/CT revealed a tendency for abnormal ^18^F-FDG accumulation and swelling of both the parotid and submandibular glands in patients with IgG4-DS, particularly in the submandibular glands. On MRI, SS had a high score regarding the findings of a salt-and-pepper appearance and/or multiple cystic areas in the parotid glands (median score 4.5). Sonography showed the highest values among the four imaging modalities for sensitivity, specificity and accuracy. There were significant differences between sonography and CT (*p* = 0.0001) and between sonography and FDG-PET/CT (*p* = 0.0058) concerning accuracy.

**Conclusions:**

Changes in the submandibular glands affected by IgG4-DS could be easily detected using sonography (characteristic bilateral nodal/reticular change) and FDG-PET/CT (abnormal ^18^F-FDG accumulation). Even inexperienced observers could detect these findings. In addition, sonography could also differentiate SS. Consequently, we recommend sonography as a modality for the screening of IgG4-DS, because it is easy to use, involves no radiation exposure and is an effective imaging modality.

## Introduction

Immunoglobulin G4 (IgG4)-related dacryoadenitis and sialadenitis (IgG4-DS), Mikulicz’s disease, has recently been recognized as being an independent entity from Sjögren’s syndrome (SS), because of its clinical and serological features [1–3]. Yamamoto et al. [2, 3] stated that these features include persistent gland swelling in IgG4-DS, while gland swelling in SS is periodic. Moreover, salivary function is either normal or improved with the administration of glucocorticoid in IgG4-DS, while it decreases and is not affected by treatment in SS. A further difference is marked elevation of serum IgG4 level in IgG4-DS, while SS show a normal serum IgG4 level. As for anti-SS-A and/or anti-SS-B antibodies, they test negative in IgG4-DS, but have a high positive rate in SS patients. Histopathologically prominent infiltration involving IgG4-positive plasmacytes has been observed using immunostaining in IgG4-DS, while no IgG4-positive plasmacytes have been seen in SS. Additionally, no punctate or globular sialectasis have been observed on sialograms in IgG4-DS, while they are generally observed in SS. Consequently, they considered IgG4-DS to be an entity independent of SS, although it is now known that there are patients who have active disease but normal serum IgG4 levels [4].

Regarding imaging diagnosis of IgG4-DS, most of the studies have been case reports, and we could only find a few which analyzed the effectiveness of the imaging modality involving ≥9 cases as follows: two studies on ^67^Ga scintigraphy; two on 2-[^18^F]-fluoro-2-deoxy-D-glucose positron emission tomography/computed tomography (FDG-PET/CT); three on sonography; and two on computed tomography (CT) and magnetic resonance imaging (MRI). Ishii et al. analyzed 13 cases with IgG4-related disease (IgG4-RD) including IgG4-DS using ^67^Ga scintigraphy, and showed that there was significant accumulation in the lacrimal glands in seven cases and in the salivary glands in seven cases [5]. In another study, they also found differences in accumulation patterns of ^67^Ga between 27 cases of sarcoidosis and 16 cases of IgG4-RD [6]. Some authors have analyzed and verified the usefulness of FDG-PET/CT for staging and follow-up, and have demonstrated abnormal ^18^F-FDG uptake in sites involved with IgG4-RD [7, 8]. Some reports have described the characteristic sonographic features of the involved glands, which might be efficient for screening IgG4-DS [9–11]. Sonography is also a useful imaging modality for the follow-up of this disease after corticosteroid therapy [9, 11]. In contrast, CT and MRI have shown enlarged glands with rather nonspecific findings regarding IgG4-DS [12, 13]; although in autoimmune pancreatitis (AIP), one of the IgG4-RD types shows very characteristic findings on both CT and MRI, which has therefore been adopted as one of the diagnostic criteria for AIP [14]. Because these studies have involved only one or two imaging modalities and comparison among modalities are not sufficient, it remains unclear as to which modality is the most efficient in detecting IgG4-DS; the differences in imaging features between IgG4-DS and SS also remain unclear.

The purposes of the present retrospective study were to analyze the imaging features of IgG4-DS using various imaging modalities, and to clarify which imaging modality is the most effective for screening this disease. We also showed the differences in imaging characteristics between IgG4-DS and SS.

## Methods

### Patients

We enrolled 39 patients with IgG4-DS (21 females and 18 males) with a mean age of 59.9 years, between 1999 and 2014. IgG4-DS was serologically and histopathologically confirmed using the following diagnostic criteria: persistent symmetrical swelling involving >2 lacrimal and major salivary glands; an elevated serum level of IgG4 (>135 mg/dl); and infiltration of IgG4-positive plasma cells (percentage of IgG4-positive cells to IgG-positive cells >40 %) on immunostaining [15]. We also analyzed 51 patients with SS (47 females and 4 males) with a mean age of 55.6 years, between 2006 and 2013. SS was diagnosed using both the revised Japanese criteria [16] and the American-European Consensus Group criteria [17]. Additionally, in 2013, 36 patients suffering from oral carcinoma, but with normal salivary glands (14 females and 22 males) with mean age of 63.3 years, were evaluated. This study design was approved by the Ethics Committee of Kyushu University, Japan, and written informed consent was obtained from all of the patients (IRB serial number: 25–287).

### Image preparation

We performed sonography on 30 patients with IgG4-DS, 38 with SS and 36 with normal salivary glands. Sonographic images were taken using a diagnostic unit (Logiq 7: GE Healthcare, Tokyo, Japan) with a center frequency of 12 megahertz (MHz). We extracted B-mode longitudinal images of both the parotid glands (parallel to the retromandibular plane) and the submandibular glands (parallel to the submandibular plane) for this study. Because insufficient Doppler images could be provided for the normal submandibular glands, we only used B-mode images, although we also recorded in the Doppler mode at the time of examination for IgG4-DS and SS. FDG-PET/CT was undertaken in 20 patients with IgG4-DS, 19 with SS and 21 with normal salivary glands. From the superimposed images, where the standardized uptake value (SUV) was reflected as graded colors, we extracted images at the parotid and submandibular gland level, not including the lacrimal gland level. Similarly, CT images at the parotid and submandibular gland level of 24, 38 and 32 patients with IgG4-DS, SS and normal salivary glands, respectively and MRI of 5, 16 and 19 patients with IgG4-DS, SS and normal salivary glands, respectively, were also extracted for this study. Regarding MRI, besides plain axial T1- and T2-weighted images, we also extracted gadolinium-enhanced T1-weighted images and coronal images of parotid and/or submandibular glands (if any were present). We did not include images of the lacrimal glands. The reasons for this were: 1) we could not undertake sonography of the lacrimal glands for safety reasons regarding the eyes; and 2) when salivary gland tumors were suspected in the patients, lacrimal glands were often not included in the scanning range on CT/MRI.

### Image analysis

Six oral and maxillofacial radiologists (with 2–26 years of diagnostic experience) randomly reviewed the arranged image sets under blinded conditions. The observation order involved sets of: 1) sonographic images; 2) FDG-PET/CT images; 3) CT images; and 4) MRI images. Observations were performed twice with a 3-week interval. Each observer was required to score the confidence rate for the presence of the characteristic imaging findings using a 5-grade rating system (1–5) in the answer sheet as follows: when written characteristic imaging findings were definitely present, 5; when written characteristic imaging findings were definitely absent, 1; presence of a swollen gland, 5; and presence of an atrophied gland, 1. After scoring each finding, diagnosis was made as normal, IgG4-DS or SS, taking into consideration all of the findings for each case.

**Fig. 1:**
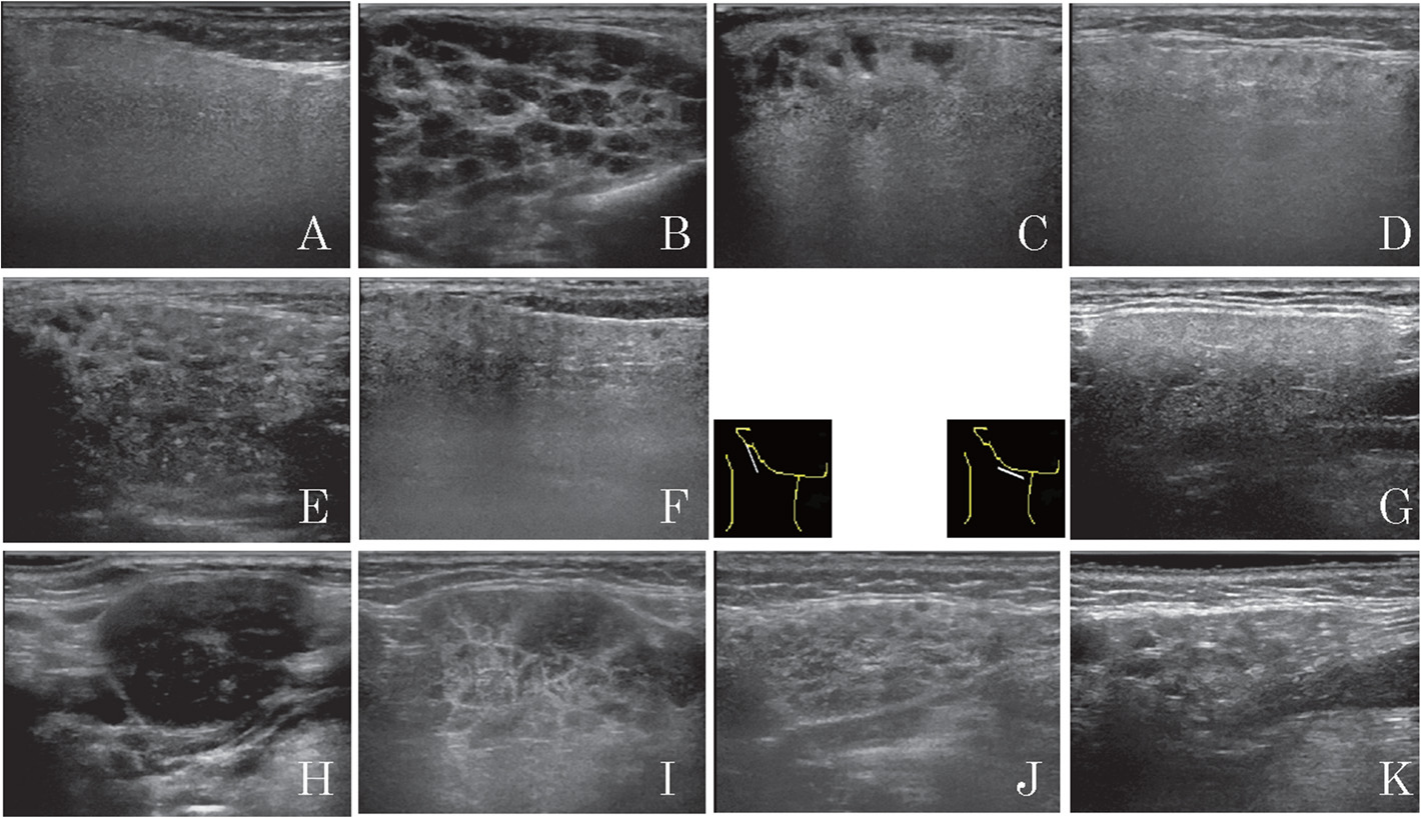
Characteristic findings on sonograms. **a** Normal parotid gland. **b-d** Parotid glands of a patient with IgG4-DS. **e, f** Parotid gland of a patient with SS. **g** Normal submandibular gland. **h, i** Submandibular glands of a patient with IgG4-DS. **j, k** Submandibular glands of a patient with SS. Multiple hypoechoic areas (**b-f, i-k**), a reticular pattern (**b, e, i**), and a nodal pattern (**h**) can be seen. Hyperechoic lines and/or spots can also observed (**b-f, i-k**). *IgG4-DS* IgG4-related dacryoadenitis and sialadenitis, *SS* Sjögren’s syndrome

Figure 1 shows the characteristic findings for IgG4-DS using sonography. They were multiple hypoechoic areas and a reticular pattern on the parotid and submandibular glands, and a nodal pattern on the submandibular glands [9–11]. The characteristic findings for SS were multiple hypoechoic areas, hyperechoic lines and/or spots, and a reticular pattern (a mixture of hypoechoic areas and hyperechoic lines) on both the parotid and submandibular glands [18, 19]. Obscuration of the gland configuration was only assessed in the submandibular glands [19].

**Fig. 2:**
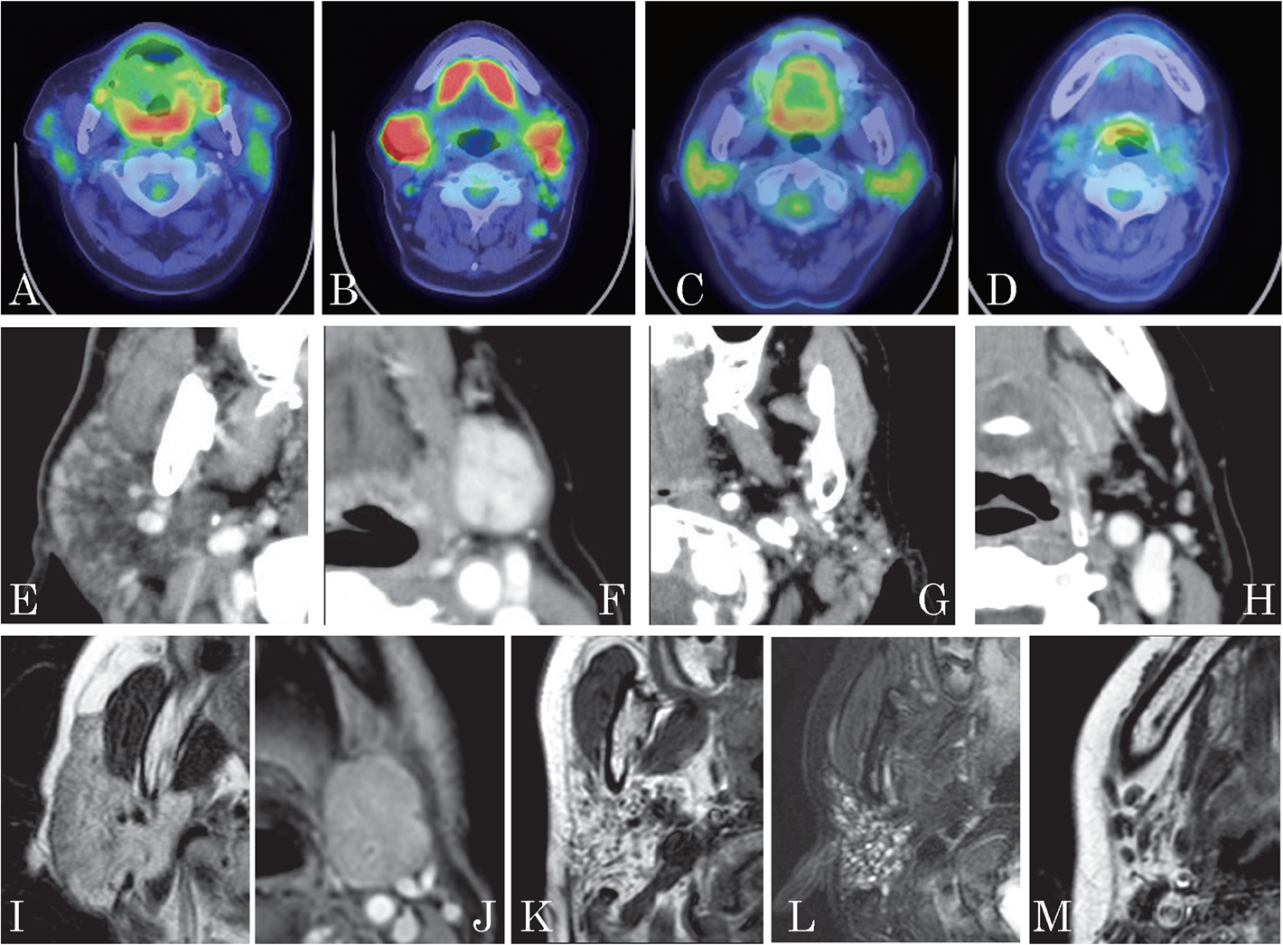
Characteristic findings on FDG-PET/CT, CT and MRI. **a-d** FDG-PET/CT, **e-h** CT and **i-m** MRI. **a, e, i** Parotid glands of a patient with IgG4-DS. **b, f, j** Submandibular glands of a patient with IgG4-DS. **c, g, k, l** Parotid glands of a patient with SS. **d, h, m** Submandibular glands of a patient with SS. FDG-PET/CT shows abnormal ^18^F-FDG accumulation in the parotid (**a, c**) and submandibular glands (**b**). A patient with IgG4-DS showing parotid (**e, i**) and submandibular gland swelling (**f, j**), superficial enhancement of the parotid glands (**e**), and a septum-like structure in the submandibular glands (**f**). A patient with SS showing atrophic parotid (**g**) and submandibular glands (**h, m**), and a salt-and-pepper appearance (**g, k, l**). It also shows dot-like calcifications on CT (**g**), and small multiple cystic areas on T2-weighted images (**l**). *FDG-PET/CT* 2-[^18^F]-fluoro-2-deoxy-D-glucose positron emission tomography/computed tomography, *CT* computed tomography, *IgG4-DS* IgG4-related dacryoadenitis and sialadenitis, *MRI* magnetic resonance imaging, *SS* Sjögren’s syndrome

On FDG-PET/CT, abnormal ^18^F-FDG accumulation in the parotid and submandibular glands was assessed as one of the characteristic findings of IgG4-DS [7, 8] (Fig. 2), when the gland exhibited bright warm colors; red corresponded to an SUV ≥6, orange corresponded to an SUV of around 5, and yellow to bright green corresponded to an SUV of around 4. Gland size was also assessed in the case of both the parotid and submandibular glands.

On CT and MRI (Fig. 2), there are nonspecific findings regarding IgG4-DS [12, 13]. From the images in the references [5, 12, 13] and empirically, we chose parotid and submandibular gland swelling and superficial enhancement of the parotid glands, and the presence of a septum-like structure on the submandibular glands as characteristic CT findings. In relation to characteristic MRI findings, we only chose gland swelling regarding IgG4-DS. In contrast, patients with SS exhibit the following characteristics: atrophic parotid and submandibular glands; heterogeneity of gland parenchyma, which is called a “salt-and-pepper appearance” [20, 21]; dot-like calcifications on CT [20]; and small multiple cystic areas, which show hyperintensity on T2-weighted images.

### Statistical analysis

We calculated the sensitivity, specificity and accuracy using the following equations: sensitivity for IgG4-DS = a/(a+d+g), sensitivity for SS = e/(b+e+h), specificity = i/(c+f+i), accuracy = (a+e+i)/(a+b+c+d+e+f+g+h+i), where a: number of patients we diagnosed with IgG4-DS and who actually had IgG4-DS, b: number of patients we diagnosed with IgG4-DS but who had SS, c: number of patients we diagnosed with IgG4-DS but who were normal, d: number of patients we diagnosed with SS but who had IgG4-DS, e: number of patients we diagnosed with SS and who actually had SS, f: number of patients we diagnosed with SS but who were normal, g: number of patients we diagnosed as normal but who had IgG4-DS, h: number of patients we diagnosed as normal but who had SS, and i: number of patients we diagnosed as normal and who were actually normal. We performed the Bartlett test for the analysis of equal variance. To determine whether or not there were significant differences between two given groups among three, we performed the Steel-Dwass test in case of unequal variance, and the Tukey-Kramer test in case of equal variance using the statistical software JMP Pro 11.0 (SAS Institute, Cary, NC, USA). *p* values <0.05 were considered significant. Intra-observer agreement rates between the repeat diagnoses (diagnosis carried out under blinded conditions and repeated after 3 weeks) were assessed with kappa values using 3 grades: 1 or 2, 3, and 4 or 5. Values <0.20 indicated poor agreement, 0.21–0.40 fair agreement, 0.41–0.60 moderate agreement, 0.61–0.80 good agreement and 0.81–1.00 excellent agreement.

## Results

Intra-observer agreement rates between the repeat diagnoses were very high. Kappa values for selecting the same diagnosis from normal, IgG4-DS or SS were 0.83 (range, 0.76–0.91) on sonography, 0.69 (range, 0.5–0.90) on FDG-PET/CT, 0.70 (range, 0.49–0.80) on CT and 0.80 (range, 0.64–0.87) on MRI. Moreover, average kappa values for each finding were 0.62 for sonography, 0.61 for FDG-PET/CT, 0.51 for CT and 0.54 for MRI; no findings showed an inverted order of scores the second time. Therefore, we have shown the results of the first diagnosis in the relevant figures.

### Analysis of sonographic findings

**Fig. 3:**
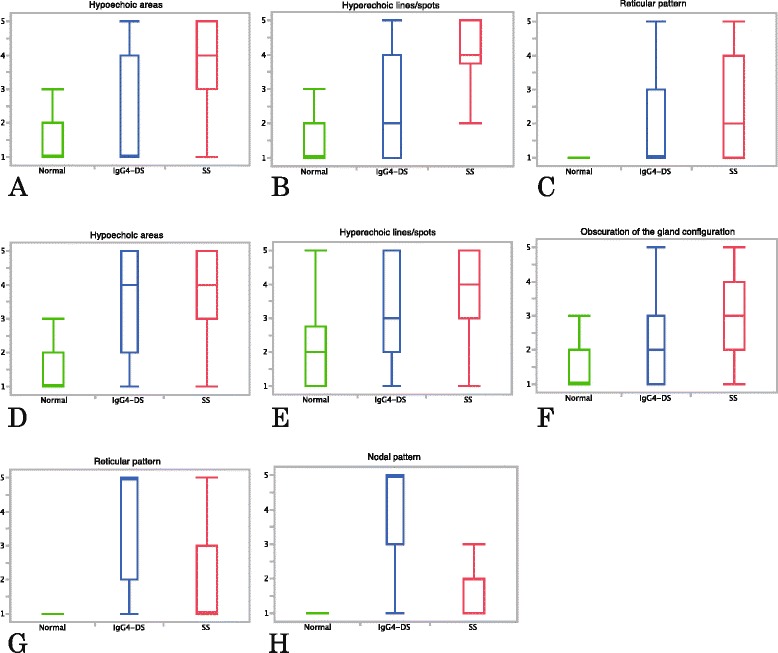
Results of sonographic analysis. Values are shown with median and quartile points for **a-c** the parotid glands and **d-h** the submandibular glands. **a** Multiple hypoechoic areas, **b** hyperechoic lines and/or spots and **c** reticular pattern in the parotid glands. **d** Multiple hypoechoic areas, **e** hyperechoic lines and/or spots, **f** obscuration of the gland configuration, **g** reticular pattern and **h** nodal pattern in the submandibular glands. Multiple hypoechoic areas (**a**) and hyperechoic lines and/or spots (**b**) in the parotid glands and obscuration of submandibular gland configuration (**f**) were primarily observed in patients with SS. Reticular and nodal patterns observed mainly in patients with IgG4-DS (**g, h**). *IgG4-DS* IgG4-related dacryoadenitis and sialadenitis, *SS* Sjögren’s syndrome

Figure 3 shows the results of sonographic analysis. In all findings, significant differences (*p* ≤0.0093) were observed between any two of the three diagnoses. The parotid glands of patients with SS mainly exhibited multiple hypoechoic areas (median score 4) and hyperechoic lines and/or spots (median score 4) (Fig. 3a and b), while the reticular pattern in the parotid glands showed overlap between IgG4-DS (median score 1) and SS (median score 2) (Fig. 3c). In the submandibular glands, overlaps were seen between IgG4-DS and SS regarding the findings of multiple hypoechoic areas (median score 4 for both IgG4-DS and SS) and hyperechoic lines and/or spots (median score 3 for IgG4-DS and 4 for SS), although significant differences (*p* = 0.0093, *p* <0.0001, respectively) were seen between the two (Fig. 3d and e). Obscuration of the submandibular gland configuration was mainly observed in SS (median score 3) (Fig. 3f). In contrast, IgG4-DS mainly exhibited reticular and nodal patterns (median score 5), and separation between IgG4-DS and the other conditions was especially good concerning the nodal pattern (median score 1 for normal glands and 2 for SS) (Fig. 3g and h). Intra-observer agreement rates between the repeat diagnoses were very high (kappa values, 0.67–0.87), which showed the nodal pattern could be easily detected.

Each case was diagnosed as normal, IgG4-DS or SS based on all of the sonographic findings. Sonographic sensitivity for detection of IgG4-DS and of SS, specificity and accuracy were 0.85, 0.80, 0.84 and 0.83, respectively.

### Analysis of FDG-PET/CT findings

**Fig. 4:**
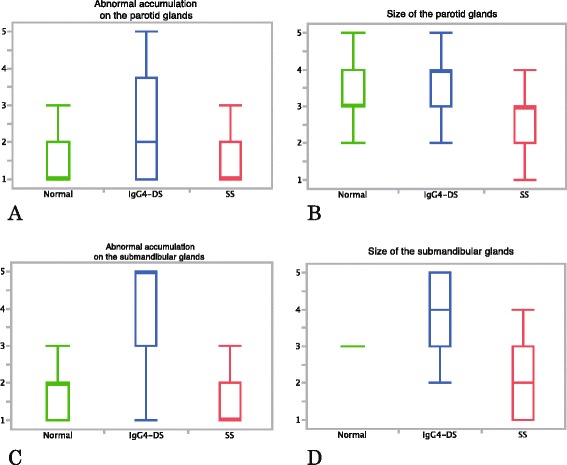
Results of FDG-PET/CT. Values are shown with median and quartile points for **a, b** the parotid glands and **c, d** the submandibular glands. **a** Abnormal accumulation of ^18^F-FDG in the parotid glands, **b** size of the parotid glands, **c** abnormal accumulation of ^18^F-FDG in the submandibular glands and **d** size of the submandibular glands. Abnormal accumulation of ^18^F-FDG and swelling of the glands in both the parotid and submandibular glands of a patient with IgG4-DS (**a-d**). Separation of IgG4-DS from the other conditions is especially good regarding the abnormal accumulation of ^18^F-FDG in the submandibular glands (**c**), and significant differences can be observed between IgG4-DS and SS (*p* <0.0001), and between IgG4-DS and normal glands (*p* <0.0001). *FDG-PET/CT* 2-[^18^F]-fluoro-2-deoxy-D-glucose positron emission tomography/computed tomography, *IgG4-DS* IgG4-related dacryoadenitis and sialadenitis, *SS* Sjögren’s syndrome

FDG-PET/CT involving patients with IgG4-DS showed a tendency for abnormal accumulation of ^18^F-FDG and swelling of both the parotid (median scores 2 and 4, respectively) and submandibular glands (median scores 5 and 4, respectively) (Fig. 4a–d). Separation between IgG4-DS and the other conditions was particularly good regarding the abnormal accumulation of ^18^F-FDG in the submandibular glands (median score 2 for normal glands and 1 for SS) (Fig. 4c). In relation to this finding, significant differences were observed between IgG4-DS and SS (*p* <0.0001), and between patients with IgG4-DS and those with normal glands (*p* <0.0001); however, this was not seen between patients with normal glands and SS (*p* = 0.1180). Intra-observer agreement rates between the repeat diagnoses were high (kappa values, 0.51–0.89). Regarding all other findings, significant differences (*p* ≤0.0049) were observed between any two of three diagnoses.

Each case was diagnosed as normal, IgG4-DS or SS based on all of the findings from FDG-PET/CT. The sensitivity for detection of IgG4-DS and of SS, specificity and accuracy using FDG-PET/CT were 0.79, 0.59, 0.81 and 0.73, respectively.

### Analysis of CT findings

**Fig. 5:**
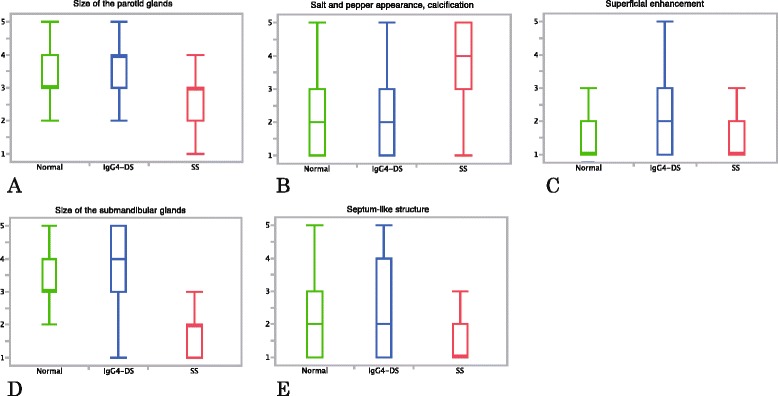
Results of CT. Values are shown with median and quartile points for **a-c** the parotid glands and **d, e** the submandibular glands. **a** Size of the parotid glands, **b** salt-and-pepper appearance and/or dot-like calcification in the parotid glands, **c** superficial enhancement of the parotid glands, **d** size of the submandibular glands and **e** septum-like structure in the submandibular glands. Patient with IgG4-DS exhibiting large parotid and submandibular glands (**a, d**). SS shows a high score in the finding of a salt-and-pepper appearance and/or dot-like calcification (**b**). *CT* computed tomography, *IgG4-DS* IgG4-related dacryoadenitis and sialadenitis, *SS* Sjögren’s syndrome

Figure 5 shows the results of CT findings concerning the parotid glands (Fig. 5a–c), and the submandibular glands (Fig. 5d and e). Patients with IgG4-DS had large parotid (median score 4) and submandibular glands (median score 4) (Fig. 5a and d). Although there were significant differences between normal glands (parotid glands, median score 3, *p* = 0.0255; submandibular glands, median score 3, *p* <0.0001), there seemed to be a similar tendency between these two. Conversely, patients with SS had small parotid (median score 3) and submandibular glands (median score 2), especially submandibular glands. Patients with SS had a high score regarding the finding of a salt-and-pepper appearance and/or dot-like calcification (median score 4) (Fig. 5b). Regarding this finding, significant differences were observed between the other two conditions (median score 2 for both normal glands and IgG4-DS) (*p* <0.0001), although this was not the case for between patients with normal glands and IgG4-DS (*p* = 0.7945). Superficial enhancement of the parotid glands (median score 2 for IgG4-DS) (Fig. 5c) and the presence of a septum-like structure in the submandibular glands (median score 2 for IgG4-DS) (Fig. 5e), which we considered were some of the characteristic findings of IgG4-DS, showed significant differences between the other two conditions (*p* <=0.0316); however, overlap was also observed and separation was not so good.

Each case was diagnosed as normal, IgG4-DS or SS based on all of the findings from CT. The sensitivity for detection of IgG4-DS and of SS, specificity and accuracy by CT were 0.64, 0.73, 0.70 and 0.70, respectively.

### Analysis of MRI findings

**Fig. 6:**
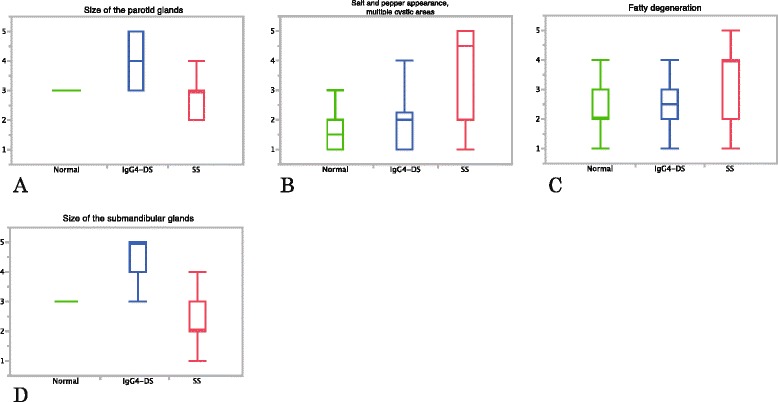
Results of MRI. Values are shown with median and quartile points for (**a-c**) the parotid glands and (**d**) the submandibular glands. **a** Size of the parotid glands, **b** salt-and-pepper appearance and/or multiple cystic areas in the parotid glands, **c** fatty degeneration of the parotid glands and **d** size of the submandibular glands. Patient with IgG4-DS showing large parotid and submandibular glands (**a, d**). SS shows small parotid and submandibular glands (**a, d**), and a high score in the finding of a salt-and-pepper appearance and/or multiple cystic areas in the parotid glands (**b**). *IgG4-DS* IgG4-related dacryoadenitis and sialadenitis, *MRI* magnetic resonance imaging, *SS* Sjögren’s syndrome

Figure 6 shows the results of MRI evaluation of the parotid glands (Fig. 6a–c), and the submandibular glands (Fig. 6d). Patients with IgG4-DS had large parotid (median score 4) and submandibular glands (median score 5) (Fig. 6a and d). These findings differed significantly between the other two conditions for both the parotid and submandibular glands (*p* <0.0001). Conversely, patients with SS had small parotid (median score 3) and submandibular glands (median score 2), especially submandibular glands; there was a very clear separation between the other two conditions. Patients with SS had a high score regarding the finding of a salt-and-pepper appearance and/or multiple cystic areas in the parotid glands (median score 4.5) (Fig. 6b); there was also a very clear separation between the other two conditions (median score 1.5 for normal glands and 2 for IgG4-DS). Intra-observer agreement rates between the repeat diagnoses were high (kappa values, 0.54–0.79) regarding this finding. In relation to this finding, significant differences were observed between the other two conditions (*p* <0.0001), although a significant difference was not seen between patients with normal glands and IgG4-DS (*p* = 0.4163). Fatty degeneration in the parotid glands, which we considered to be one of the characteristic findings of SS, showed significant differences between the other two conditions (*p* <=0.0061); however, overlap was also observed and separation was not so good.

Each case was diagnosed as normal, IgG4-DS or SS based on all of the findings. The sensitivity for detection of IgG4-DS and of SS, specificity and accuracy using MRI were 0.80, 0.67, 0.81 and 0.76, respectively.

### Comparison of the four imaging modalities

**Fig. 7:**
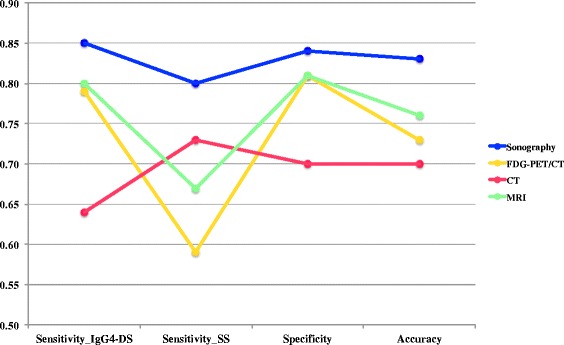
Sensitivity, specificity and accuracy of the four imaging modalities. Values are the average for all observers of each modality. Sonography showed not only the highest accuracy, but also the highest sensitivity for both IgG4-DS and SS, and the highest specificity among the modalities. There were significant differences between sonography and CT (*p* = 0.0001) and between sonography and FDG-PET/CT (*p* = 0.0058) regarding accuracy. *CT* computed tomography, *FDG-PET/CT* 2-[^18^F]-fluoro-2-deoxy-D-glucose positron emission tomography/computed tomography, *IgG4-DS* IgG4-related dacryoadenitis and sialadenitis, *MRI* magnetic resonance imaging, *SS* Sjögren’s syndrome

Figure 7 shows the sensitivity, specificity and accuracy of the four imaging modalities; sonography showed the highest levels. Regarding the sensitivity of IgG4-DS, there were significant differences between CT and the other three modalities (*p* <0.001). Concerning the sensitivity of SS, there were significant differences between sonography and FDG-PET/CT (*p* = 0.0004) and between CT and FDG-PET/CT (*p* = 0.0180). In relation to specificity, there were significant differences between sonography and CT (*p* = 0.0463). Regarding the accuracy, there were significant differences between sonography and CT (*p* = 0.0001) and between sonography and FDG-PET/CT (*p* = 0.0058); however, there were no significant differences between sonography and MRI (*p* = 0.0796).

## Discussion

For screening IgG4-DS, the nodal pattern of the submandibular glands on sonograms and the abnormal accumulation of ^18^F-FDG in the submandibular glands on FDG-PET/CT were very effective. However, the effective findings regarding the screening of SS were a salt-and-pepper appearance and/or multiple cystic areas in the parotid glands on MRI. Taking into consideration high sensitivity for both IgG4-DS and SS, and high specificity in addition to high accuracy, the most effective imaging modality was sonography.

We did not include images of the lacrimal glands. Even if they were included, we could detect the affected lacrimal glands as a nodal pattern on sonograms, as abnormal ^18^F-FDG accumulation on FDG-PET/CT, and as abnormal swelling on CT and MRI. Consequently, the abnormal findings of images of the lacrimal glands would raise the accuracy of all modalities equally, and the effectiveness of each modality would not change appreciably.

### Sonography

Sonography provided useful information regarding the screening of IgG4-DS. The nodal and reticular patterns, often with normal parenchyma surrounding the affected region and located bilaterally, were easily detected in the submandibular glands even by inexperienced observers. When the sublingual glands are affected, they also show a nodal and/or reticular pattern on sonograms (data not shown); therefore, it would be easier to make an accurate diagnosis. Using sonography, not only IgG4-DS but also SS can be detected. Multiple hypoechoic areas and hyperechoic lines and/or spots in the parotid glands achieved high scores. These findings might be misleading, because IgG4-DS sometimes exhibits similar findings. However, hypoechoic areas of IgG4-DS were observed in the normal parotid parenchyma without a reduction in the echo intensity level and heterogeneity. Moreover, IgG4-DS mainly affects the submandibular glands, and most of the cases present with normal parotid glands [9]. Conversely, SS shows atrophic changes in both the parotid and submandibular glands. Therefore, the combination of parotid and submandibular gland findings could lead to accurate diagnosis. In the present study we only analyzed B-mode sonograms. If Doppler images are added, it can make differentiation between IgG4-DS and SS much easier. The nodal and reticular patterns of IgG4-DS show high vascularity [9], while SS exhibits small dot-like vascularity in the parotid glands [22]. When the IgG4-DS patients had shown submandibular gland swelling and salivary gland tumors had been suspected, sonography revealed nodal and/or reticular changes on both sides, and tumorous lesions were easily ruled out.

### FDG-PET/CT

FDG-PET/CT showed abnormal ^18^F-FDG accumulation in the glands affected with IgG4-DS. However, this imaging modality could not differentiate between normal glands and SS. Patients with SS do not undergo FDG-PET/CT, unless they have other malignant diseases. Our cases involving FDG-PET/CT were patients with SS who were partly suspected of having malignant lymphoma. When patients actually had lymphoma, the SUV was high and we could not differentiate the disease from IgG4-DS. Conversely, very severe SS with atrophic submandibular glands exhibited a very low SUV both in the parotid and submandibular glands. In contrast, SS patients in the lower stages (their sialography showed punctuate or globular patterns) had a rather high SUV (SUV; 3–4). This finding was in accordance with a report by Cohen et al. [23]. Normal salivary, sublingual, submandibular and parotid glands sometimes show high SUVs. Because Nakamoto et al. have reported that the intensity of ^18^F-FDG uptake in the salivary glands is variable [24], we need to carefully differentiate IgG4-DS from normal variances.

### CT and MRI

Nodal changes in IgG4-DS detected using sonography were not clearly observed on CT or MRI. In some cases CT and MRI displayed superficial enhancement in the parotid glands of IgG4-DS. However, unspecific swelling of the parotid and submandibular glands was observed in general. Most of the patients with normal salivary glands, that were large in size, were misdiagnosed as IgG4-DS. Some of our cases showed slightly low signal intensity on T2-weighted images on MRI; however, they were not significant. These results were not in accordance with the very low signal intensity on T2-weighted images [12, 13]. This may be because of the limited number of our MRI of the IgG4-DS patients. Diffusion-weighted images might differentiate nodal changes regarding IgG4-DS more clearly. SS, however, showed a characteristic salt-and-pepper appearance on CT and MRI. When this appearance was not pronounced, inexperienced observers could not detect it on CT.

Two things are important in diagnosing IgG4-DS. The first is to differentiate malignant lymphoma. Malignant lymphoma sometimes affects salivary glands, and can appear in bilateral glands, which mimic IgG4-DS [25]. To make a final diagnosis, biopsy is recommended. Second, IgG4-DS does not always occur simultaneously in both the lacrimal and salivary glands. Even if the findings are indefinite at the time of examination, follow-up is necessary when IgG4-DS is suspected.

## Conclusions

Changes in the submandibular glands affected by IgG4-DS, which often occur bilaterally, could be easily detected using sonography as a result of characteristic bilateral nodal/reticular changes and by FDG-PET/CT because of abnormal ^18^F-FDG accumulation. Even inexperienced observers could detect these findings. In addition to IgG4-DS, sonography could also differentiate SS. Therefore, we recommend sonography as a modality for the screening of IgG4-DS, because it is easy to use, does not involve radiation exposure and is an effective imaging modality.

## Abbreviations

AIP: autoimmune pancreatitis
CT: computed tomography
FDG-PET/CT: 2-[^18^F]-fluoro-2-deoxy-D-glucose positron emission tomography/computed tomography
IgG4: immunoglobulin G4
IgG4-DS: IgG4-related dacryoadenitis and sialadenitis
IgG4-RD: IgG4-related disease
MHz: megahertz
MRI: magnetic resonance imaging
SS: Sjögren’s syndrome
SUV: standardized uptake value
